# Connexin43 Forms Supramolecular Complexes through Non-Overlapping Binding Sites for Drebrin, Tubulin, and ZO-1

**DOI:** 10.1371/journal.pone.0157073

**Published:** 2016-06-09

**Authors:** Cinzia Ambrosi, Cynthia Ren, Gaelle Spagnol, Gabriel Cavin, Angela Cone, Elena E. Grintsevich, Gina E. Sosinsky, Paul L. Sorgen

**Affiliations:** 1 National Center for Microscopy and Imaging Research, Center for Research in Biological Systems, University of California La Jolla, La Jolla, California, United States of America; 2 Department of Neurosciences, University of California La Jolla, La Jolla, California, United States of America; 3 Department of Chemistry and Biochemistry, University of California Los Angeles, Los Angeles, California, United States of America; 4 Department of Biochemistry and Molecular Biology, University of Nebraska Medical Center, Omaha, Nebraska, United States of America; Emory University School of Medicine, UNITED STATES

## Abstract

Gap junctions are membrane specialization domains identified in most tissue types where cells abut each other. The connexin channels found in these membrane domains are conduits for direct cell-to-cell transfer of ions and molecules. Connexin43 (Cx43) is the most ubiquitous connexin, with critical roles in heart, skin, and brain. Several studies described the interaction between Cx43 and the cytoskeleton involving the actin binding proteins Zonula occludens (ZO-1) and drebrin, as well as with tubulin. However, a direct interaction has not been identified between drebrin and Cx43. In this study, co-IP and NMR experiments were used to demonstrate that the Cx43-CT directly interacts with the highly conserved N-terminus region of drebrin. Three Cx43-CT areas were found to be involved in drebrin binding, with residues 264–275 being critical for the interaction. Mimicking Src phosphorylation within this region (Y265) significantly disrupted the interaction between the Cx43-CT and drebrin. Immunofluorescence showed colocalization of Cx43, drebrin, and F-actin in astrocytes and Vero cells membrane, indicating that Cx43 forms a submembrane protein complex with cytoskeletal and scaffolding proteins. The co-IP data suggest that Cx43 indirectly interacts with F-actin through drebrin. Along with the known interaction of the Cx43-CT with ZO-1 and tubulin, the data presented here for drebrin indicate non-overlapping and separated binding sites for all three proteins for which simultaneous binding could be important in regulating cytoskeleton rearrangements, especially for neuronal migration during brain development.

## Introduction

Gap junction channels provide a pathway for direct cell-to-cell communication between adjacent cells. These channels are involved in a number of biological functions such as electrical conduction, embryogenesis, and cell growth [1]. In order to assure proper regulation of intercellular communication, gap junction proteins interact with several cytosolic proteins that serve as part of a larger cellular signaling platform called “the nexus” [2, 3]. Gap junctions are formed by the apposition of connexons from adjacent cells, where each connexon is formed by six connexin proteins [4]. Though the twenty-one connexin isoforms share significant sequence homology, the major divergence in primary structures occurs in the cytoplasmic loop and carboxyl-terminal (CT) domains. [5]. Nuclear magnetic resonance (NMR) studies and one crystallographic structure of CT connexin peptides have shown that this domain contains the most flexible sequences that bind to other proteins inducing small, localized conformational rearrangements [6–13].

To study connexin structure and interaction aspects, we chose to examine Connexin43 (Cx43), because it is the most ubiquitous and highly expressed connexin, with widespread tissue expression and critical roles in heart, skin, and brain. The Cx43-CT interacts with a number of proteins which include the proto-oncogene Src, Zonula occludens 1 (ZO-1), α- and β-tubulin, serine kinases, and phosphatases [9, 12, 14–25]. While Cx43 is involved in neuronal migration during brain development, expression is not found in adult neurons. However, the protein level of Cx43 expression remains high in adult astrocytes. During brain development, neural cells extensively couple through Cx43 gap junctions [26, 27]. Cx43 has been localized at the points of contact between migrating neurons and radial glial fibers during development [28, 29], suggesting its importance in neuronal migration [30]. In developing rat cortex, radial glial cells with Cx43 expression levels knocked-down are unable to migrate to the cortical plate and remain in the intermediate zone. Further studies showed that Cx43 is involved in neuronal migration by mediating cells adhesion rather than by forming intercellular channels [29]. Little is known about the way gap junction adhesions interact with the internal cytoskeleton [30]. Cx43 has been described to bind several actin-interacting proteins including vinculin, ZO-1, and drebrin E [15, 20, 31].

*Drebrin* is a *d*evelopmentally *re*gulated *b**r*ain prote*in* that was first isolated from chick (*Gallus gallus*) embryos [32, 33]. Three isoforms were found generated by RNA alternative splicing from a single gene, two embryonically expressed (E1 containing no insert and E2 with a 43 amino acid insert) and one expressed in adult cells (drebrin A containing both the 46 and 43 amino acid inserts) [34–36]. A fourth drebrin isoform, called s-drebrin A, has been identified in post-natal murine brain, and it is a C-terminal truncated version of drebrin A accompanied with a S368R substitution [37]. Drebrin A is highly enriched in dendritic spines, where it regulates their shapes and densities, via the rearrangement of cytoskeletal actin filaments [38–41]. Recent studies have demonstrated that drebrin A binds to F-actin with a stoichiometry of one to five protomers [16]. The ability of drebrin A to quickly re-shape cellular membranes explains why this protein is involved in the change of the morphology and density of dendritic spines [42–46]. Drebrin A expression knocked-down in the whole rat brain resulted in memory defects, sensorimotor gating, and cognitive function [47]. In addition, a precipitous decrease of drebrin A has been found in dendritic spines before loss of synapses in people with mild cognitive impairment [48], Alzheimer’s disease [49–51], and Down syndrome [51]. Complete drebrin A loss causes memory deficit [47, 52], while drebrin E up-regulation has been linked to various carcinomas [34, 53].

Drebrin E and Cx43 were shown to colocalize in contact zones of astrocyte plasma membrane and the interaction is required to maintain Cx43 gap junctions in their functional state [15]. Drebrin may utilize both the N-terminal actin depolymerizing factor and the proline-rich binding domains to interact simultaneously with cytosolic tails of transmembrane proteins and to serve as linkage points to the actin cytoskeleton. As originally described by Butkevich et al. [15], drebrin E is a novel interaction partner of the Cx43-CT. Their studies in Vero cells (a kidney epithelial cell line, extracted from an African green monkey) showed colocalization of Cx43 and drebrin E at the plasma membrane in regions of cell-cell contact and Cx43 still colocalized with drebrin E after drug induced actin depolymerization [15]. Altogether the data indicate drebrin E and Cx43 are present in the same complex, independent of actin.

Here, we describe experiments using a co-immunoprecipitation (co-IP) strategy and changes in NMR spectra of the Cx43-CT to confirm the existence of a direct interaction between Cx43-CT and drebrin at areas in the Cx43-CT sequence spatially distinct from tubulin and ZO-1. We specifically identified the N-terminus of drebrin (residues 1–300) as the fragment that binds to the Cx43-CT and three specific Cx43-CT aminoacidic sequences are affected by this interaction. Of note, mammalian embryonic and adult drebrin isoforms share a highly conserved sequence in this N-terminal region [34]. We also identified that the association between the Cx43-CT and F-actin is likely mediated by drebrin in a model similar to E-cadherin/B-catenin/actin complex, as identified by other research groups [15, 54]. In addition, mimicking phosphorylation of the Cx43-CT residue Y265 by Src inhibits the binding of Cx43 and drebrin. Altogether, we propose a model illustrating how Cx43 links to the cytoskeleton through different proteins: tubulin (microtubules), ZO-1 (actin), and drebrin (actin).

## Material and Methods

### DNA constructs

The following vectors were used for protein expression in this study: 1) Drebrin A 1–300 (Drb^1-300^) was created by introducing a stop codon after residue 300 in full-length drebrin A DNA subcloned into pGEX-4T1 [16]; 2) His-tagged Cx43-CT residues 234–382 (His-Cx43-CT^234-382^) was cloned into pET-14b. The molecular weight of this fragment untagged is ~19.6 kDa; 3) GST-tagged Cx43CT residues 316–382 (GST-Cx43-CT^316-382^) was cloned into pGEX-6P-2. The construct has a molecular weight of ~35.5 kDa; 4) GST-Cx43-CT^240-382^ wt and deletions were cloned into pGEX-TK2. The CT fragment deleted at amino acids 261–280 starts at residue 244 and a Met was inserted between the GST tag and the CT (molecular weight of ~41 kDa). All the other deletion fragments start at V240 with molecular weights of ~42 kDa; 5) GST-ZO-1^PDZ2^ (residues 186–262 of human ZO-1) was cloned into pGEX-6P-2. The resulting construct is ~43 kDa.

### Protein purification

BL21 DE3 *Escherichia coli* (New England Biolabs, Ipswich, MA) cells transformed with Cx43-CT fragments or the PDZ2 domain of ZO-1 were grown at 37°C to an OD_600_ of >0.6 in Luria-Bertani medium containing ampicillin. Expression induction was performed by adding 1 M IPTG to the media. After overnight growth, cultures were centrifuged for 10 minutes at 1700 x G at 4°C. The cell pellet was lysed with a buffer containing protease inhibitors (Sigma, St. Louis, MO), 0.5M EDTA, 0.2M PMSF, Triton X-100, and 1X PBS and sonicated for 30 seconds at power 80. The resulting lysate was centrifuged at 18,500 x g for 30 minutes at 4°C. For GST-tagged proteins, the supernatant was incubated overnight with Pierce glutathione agarose (Thermo Scientific, Rockford, IL). For the His-tagged Cx43-CT fragment, the supernatant was incubated overnight with Ni^2^+-nitrilotriacetate (NTA) agarose (Qiagen, Hilden). Resins were then washed with 1X PBS. GST-tagged proteins were eluted with 50mM Tris (pH 8.0) containing 10 mM reduced glutathione. His-tagged proteins were eluted with 50 mM Tris (pH 8.0) containing 300 mM L-Histidine and then untagged using TEV protease (Cat. # T4455) purchased from Sigma-Aldrich (St. Louis, MO). The GST-tag of the Cx43CT^234-382^ and Cx43CT^234-382^ Y265D constructs used in the NMR experiments was cleaved by incubating the beads with Turbo 3C (Accelagen, San Diego, CA).

The Drb^1-300^ construct was purified according to the protocol reported in Grintsevich et al. [16]. Briefly, the DNA construct encoding Drb^1-300^ was expressed in Rosetta cells (Novagen, Darmstadt, Germany). Cells were grown at 37°C until OD_600_ = 0.6–0.8, following the induction with 0.2 mM IPTG for 4–5 hours. The construct was purified on glutathione-agarose column. The GST tag was digested and captured by using a Thrombin kit (Cat. # 69022–3; Novagen, Darmstadt, Germany).

Additionally, bovine tubulin lyophilized (Cat. # TL238-A), porcine brain tubulin rhodamine (Cat. # TL590M-A), and rabbit skeletal muscle actin (Cat. # AKL99-A) purified proteins were purchased from Cytoskeleton Inc. (Denver, CO). Transferrin (Tfn) (Cat. # 90190-100MG) was purchased from Sigma-Aldrich (St. Louis, MO).

### Co-immunoprecipitation

Antibodies: mouse anti-Cx43 IF1 (that binds aa 360–382, from Paul Lampe lab, see [55]), mouse anti-Cx43 252–270 (m252-270, Cat. # 610061; BD biosciences), mouse anti-actin (Cat. #MA1-744; Thermo scientific, Rockford, IL), rabbit anti-GST (Cat. # G7781; Sigma-Aldrich, St. Louis, MO). Pierce protein A agarose (Cat. # 20333; Thermo Scientific, Rockford, IL) beads were washed with 1% Bovine Serum Albumin (BSA) in 1X PBS and 10% SDS. The agarose beads were then washed three-four times in 1X PBS to remove the SDS and binding solution was added (1% BSA in 1X PBS). 4 μl of the antibody was added to the agarose beads. The antibody was allowed to bind to the resin at 4°C for 4 hours. After incubation, the agarose beads were washed four times with cold 1X PBS. More binding solution and 50 μg (for the overloading condition) or 20 μg (for the standard condition) of bait protein (CT fragments or deletions or F-actin) and prey protein (tubulin, GST-ZO-1^PDZ2^, Drb^1-300^, and transferrin) were added to the agarose beads. After overnight incubation, the agarose beads were washed four times with cold 1X PBS and 20 μl of 5% BME Novex Tricine loading sample buffer (Life Technologies, Inc., Carlsbad, CA) was added to each sample.

### Gel Electrophoresis, Western blot, imaging, density determination, and statistical analysis

Samples were boiled for 5 minutes and ran on a 4–20% Tris-Glycine polyacrylamide gel (Life Technologies, Inc., Carlsbad, CA). Polyacrylamide gels were then fixed and stained with SYPRO-Ruby solution (SR) (Life Technologies, Inc., Carlsbad, CA). For Western blot, after electrophoresis the protein bands were transferred to PVDF membrane using the iBlot system (Life Technologies, Inc., Carlsbad, CA). The primary antibody was diluted in LI-COR blocking buffer (Cat. # 927–40000) and fluorescent secondary antibodies (IRDye 800 CW Goat anti-Rabbit IgG, Cat. # 926–32211 and IRDye 680RD Goat anti-Mouse IgG, Cat. # 926–68070) were used for band detection. We performed analysis of the fluorescent protein bands by using a Li-COR Odyssey Imager Fc Instrument (LI-COR Biosciences, Lincoln, NE). Tubulin-rhodamine bands were detected by using the Gel Logic 200 imaging system (Carestream Health Inc., Rochester, NY). Bands analysis and measurements were performed using the Image-Studio software (LI-COR Biosciences, Lincoln, NE) program and Excel for averaged values and standard deviation determination. For the definitive analysis at least four different replicates were chosen based on high consistency. We used the Prism 6 software package (GraphPad, La Jolla CA) to test for statistical significance levels between samples. In particular, an unpaired *t*-test for pair-wise comparisons was applied to all the studies, given the homogenous standard deviations.

### NMR measurements

NMR data were acquired at the University of Nebraska Medical Center’s NMR Facility using a 600 MHz Varian INOVA NMR Spectrometer outfitted with a cryo-probe. NMR spectra were processed and phased using NMRPipe and NMRDraw, and analyzed using NMRView. Binding isotherms were obtained from gradient-enhanced two-dimensional ^15^N-HSQC experiments, acquired with 1024 complex points in the direct dimension and 128 complex points in the indirect dimension. Sweep widths were 8,000 Hz in the ^1^H dimension and 1,720 Hz in the ^15^N dimension. ^15^N-labeled Cx43-CT domain (WT or Y265D mutant) in 1X PBS (pH 7.5) was maintained constant at 50 μM while adding increasing amount of Drb^1-300^. Dissociation constants (*KD*) were calculated by nonlinear fitting of the decrease in signal intensity of at least three residues (*KD* ± standard deviation of the mean) using GraphPad Prism 5.0 (GraphPad Software, La Jolla, CA).

### Immunolabeling of rat brain slice, cultured astrocytes, and Vero cells

One female Sprague Dawley rat five weeks old was used for our rat brain mosaic. The rat was anesthetized with an overdose of Nembutal (10 mg/100 gm body weight) and perfused transcardially with oxygenated Ringer's solution at 37°C (0.79% NaCl, 0.038% KCl, 0.020% MgCl2·6H2O, 0.018% Na2HPO4, 0.125% NaHCO3, 0.030% CaCl2·2H2O, 0.20% dextrose, and 0.020% xylocaine) for ∼2 min, followed by 0.1 M PBS, pH 7.4, containing 4% paraformaldehyde (37°C). The fixative was perfused through the body for 10 minutes, the brain was removed and cut on a vibratome into 100 μm thick sagittal slices. The slices were stored in ice-cold 1X PBS and used for the immunolabeling.

Immunolabeling was performed following the standard procedure [56] using: primary anti-drebrin antibody (M2F6 Cat. #12350, Abcam Inc, Cambridge, MA; RRID:AB_299034) and FITC for the secondary antibody; Rabbit polyclonal antibody anti-Cx43 (C6219 Sigma-Aldrich, St. Louis, MO) and CY5 for secondary antibody; anti-glial fibrillary acidic protein (GFAP) antibody or chicken anti-GFAP in blue (Neuromics, Catalog # CH22102) and Rhodamine Red X as secondary antibody.

Primary human astrocytes were purchased from Science Cells (Carlsbad, CA; Cat. # 1800) and Vero cells from ATCC (Manassas, VA). Both cell lines were cultured following a protocol provided and recommended by the manufacturing company. Immunolabeling of cultured astrocytes and Vero cells was performed following a procedure similar to the rat brain slice, but different secondary antibodies were used: for drebrin, Rhodamine Red X; for Cx43, CY5 and for Phalloidin, donkey anti-rabbit FITC or donkey anti-chicken FITC and stained with phalloidin-Rhodamine Red X. FITC secondary antibody was used. Channel colors were changed using image J to match up every figure as for the resulting drebrin in green, Cx43 in red and actin or chicken anti-GFAP in blue (Neuromics, Catalog # CH22102).

### Imaging of immunolabeled rat brain slices, cultured astrocytes, and Vero cells

In order to generate the rat brain mosaic, the immune-labeled rat brain slice was imaged using an Olympus Fluoview 1000. The resolution of the images was 512x512 pixels, with 1.2419 microns/pixel. A 10x no oil objective was used (at 2x zoom), and 496 total tiles (fields of view) were acquired with 25 z-slices on every field of view (5 microns of depth per slice). All tiles were maximum intensity projected to produce a single image. For the inset high-resolution images, an Olympus Gemini was used at different settings (60x oil with 1.5x zoom; 0.1380 microns/pixel) and z-stacks were acquired (5 microns per slice, 15 slices). A single slice was chosen from the stack to be used in the inset. The cultures cells were imaged using an Olympus Gemini; the objective was 60x oil at 3x zoom (0.069 microns/pixel); single slices were chosen from z-stacks for the individual images.

## Results

### Cx43 and drebrin colocalize in brain tissue and in cell models

**Fig 1:**
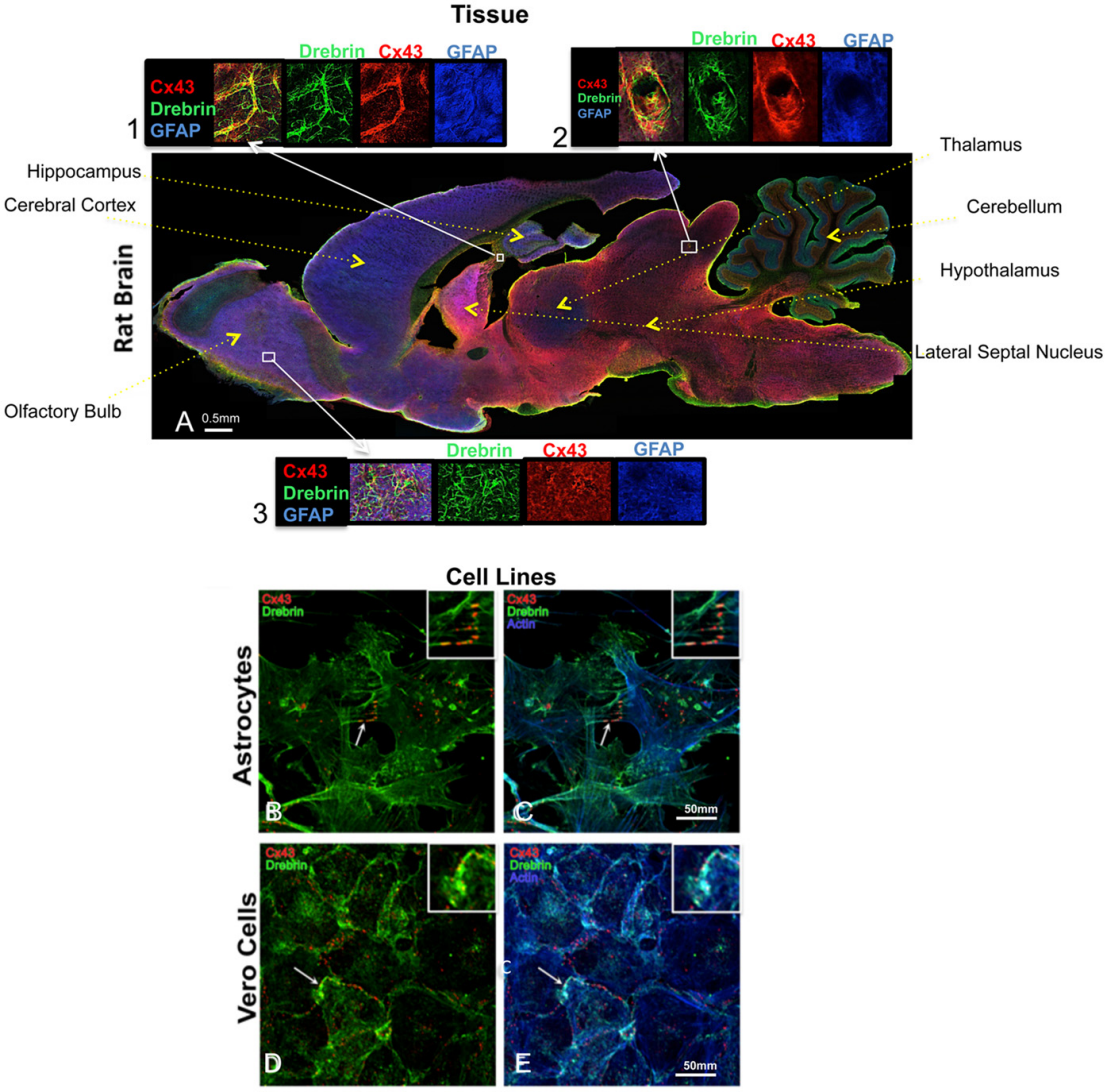
Cx43 and drebrin colocalization analysis in brain and cellular models. (A) Rat brain transversal slice mosaic shown after multiple immunolabeling with antibodies anti-Cx43 (*red*), anti-drebrin (*green*), and anti-GFAP (*blue*) as astrocytes marker. White boxes localize the area enlarged in insets 1, 2, and 3 (six fold enlargement). Colocalization of drebrin and Cx43 (*yellow*) is especially noticeable around the blood vessels (inset 2) and in regions rich of astrocytes (insets 1 and 3). The different regions of the brain were labeled. Cultured astrocytes (B and C) and Vero cells (D and E) were immunolabeled with anti-Cx43 (*red*), anti-drebrin (*green*), and anti-actin (*blue*). White arrows indicate zones of colocalization of Cx43, drebrin and actin that were enlarged in the insets (white boxes, three fold enlargement).

Previously published data showed that Cx43 and drebrin colocalize in primary mouse astrocytes and Vero cells [15]. To determine whether Cx43 and drebrin colocalize in brain tissue, a transversal slice of an adult rat (5 week old) was immunostained with anti-GFAP (which exclusively binds to protoplasmic and fibrous astrocytes in the CNS [57]) (Fig 1A). At low resolution, several brain regions show strong drebrin expression [58]. These include the cerebellum, as well as every region rich in mature neurons and astrocytes. Cx43 and drebrin colocalization can be observed in small yellow dots within the astrocytes present throughout the imaged brain and enlarged in three insets (Fig 1A, insets 1, 2 and 3). However, the most obvious colocalization of Cx43 and drebrin is in the astrocytes and endothelial cells that surround the blood vessels forming the blood-brain-barrier (ovular shapes, Fig 1A, inset 2). This colocalization is consistent with the neural transcriptional database, which shows the expression of both Cx43 and drebrin in astrocytes and endothelial cells [59].

Since drebrin is an F-actin-binding protein, the primary human astrocyte cells and Vero cell line used to determine Cx43 and drebrin colocalization [15] were also immunostained with F-actin. Immunofluorescence confirmed Cx43 and drebrin overlapping in astrocyte protrusions at the plasma membrane (Fig 1B, white arrow and inset), in addition to showing colocalization of Cx43 with drebrin and F-actin (Fig 1C, white arrow and inset). A similar colocalization between the three proteins can be observed in Vero cells (Fig 1D and 1E).

### N-terminal region of drebrin mediates the interaction between Cx43 and F-actin

**Fig 2:**
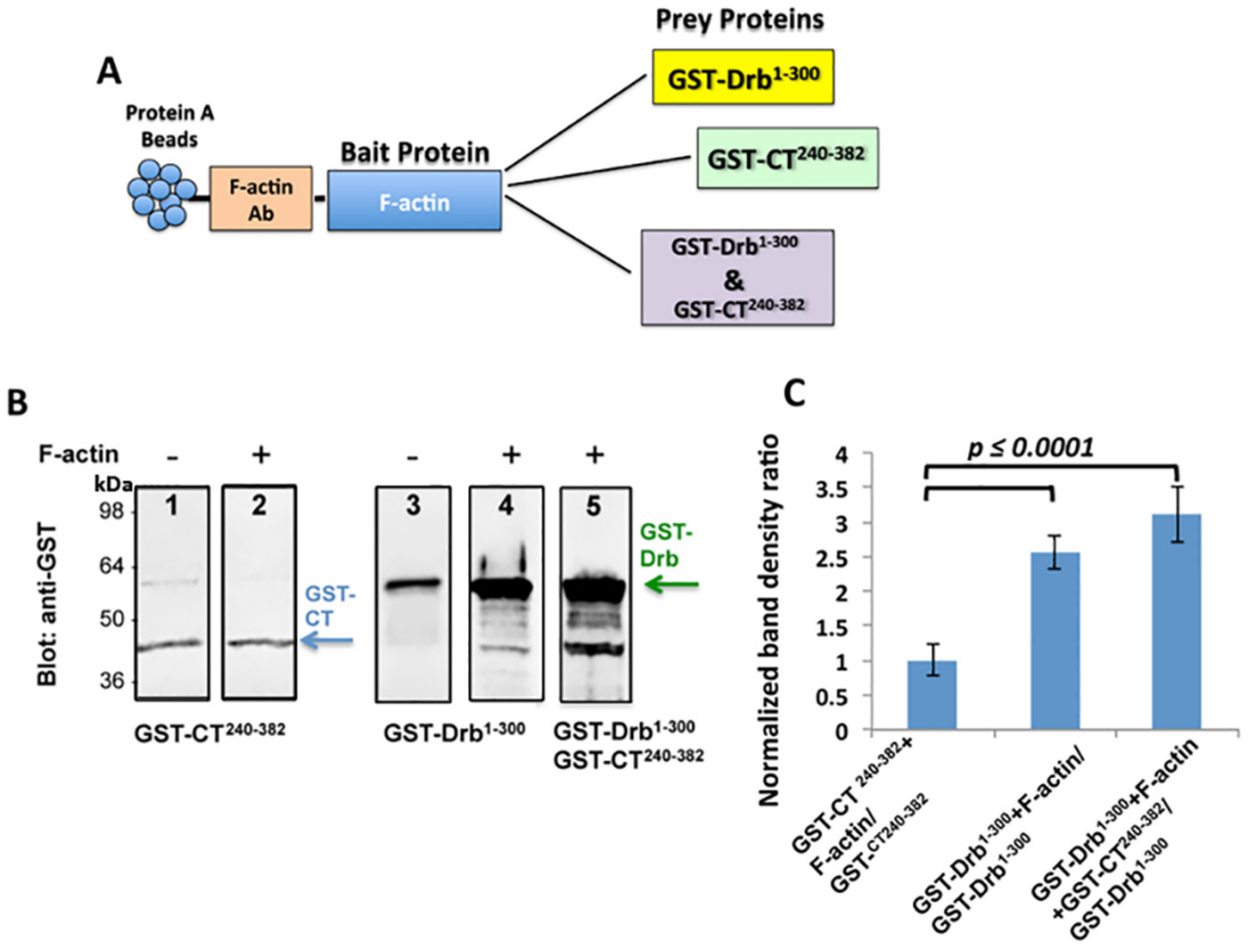
Co-IP experiment confirms that Cx43 interacts with F-actin through drebrin binding. (A) Schematic of the co-IP strategy: F-actin antibody was bound on protein A agarose beads to capture F-actin, serving as the “bait” protein. GST-Cx43-CT^240-382^ and GST-Drb^1-300^ purified constructs were used as “prey” proteins. (B) Representative lanes of Western blots chosen as best example from four independent experiments performed with a rabbit anti-GST antibody. The arrows indicate the GST-Cx43-CT^240-382^ (*blue*) and GST-Drb^1-300^ (*green*) bands obtained with or without the addition of F-actin (lanes 2, 4, and 5, and 1 and 3 respectively). (C) Quantification of the data shown in (B). GST-Cx43CT^240-382^ or GST-Drb^1-300^ co-IP band intensities were normalized to the band obtained without the “bait” protein (F-actin). The p value is the result of the T-test ran to confirm significant difference between the two samples. Error bars represent the standard deviation between four replicates.

Based on previous studies which show that the Cx43-CT binds multiple protein partners [6–13], we performed a co-IP experiment to test for any direct interaction between the Cx43-CT and the cytoskeletal protein F-actin. As described in the schematic in Fig 2A, for the co-IP strategy, we used anti-F-actin antibody bound to protein A agarose beads and added purified F-actin as a “bait” to form a complex with the antibody. Prior work with an N-terminal fragment of drebrin (Drb^1-300^) indicated that this construct contains the actin-binding domain [16]. We added the GST-Drb^1-300^ and GST-Cx43-CT^234-382^ to the protein A agarose beads as “prey” proteins. As negative controls (for detecting non-specific binding), we used protein A agarose beads bound to anti-F-actin antibody mixed with “prey” proteins in absence of any “bait”. After the pull-down, we analyzed the resulting binding through a Western blot performed by utilizing a rabbit anti-GST primary antibody.

Fig 2B reports the best representative and consistent Western blot lanes (out of four different experiments) that show no difference in the band density between GST-Cx43-CT^240-382^ with and without F-actin as “bait” on the protein A agarose beads (Fig 2B, lanes 1 and 2), as confirmed by the quantification (Fig 2C). Lanes 3 and 4 in Fig 2B represent the difference of GST-Drb^1-300^ pulled-down without and with F-actin as “bait” respectively. As detectable by Western blot and as confirmed by the band density measurements in Fig 2C, the amount of Drb^1-300^ binding to the protein A agarose beads in the presence of actin is about 2.5 fold more abundant than when there is no F-actin bound to the protein A agarose beads, confirming previously published data [23]. When we mixed together both GST-Drb^1-300^ and GST-Cx43-CT^234-382^ with F-actin (Fig 2B, lane 5), the co-IP resulted in a GST-Drb^1-300^ band density similar to Fig 2B, lane 4. The graph in Fig 2C confirms the similarity of the normalized band densities for GST-Drb^1-300^ when mixed only with F-actin and when added to F-actin and GST-Cx43-CT^240-382^. These data suggest that Cx43-CT does not contain an F-actin binding site, excluding the background. However since drebrin does bind to F-actin [16], we hypothesize that drebrin plays a role as mediator in the interaction between Cx43 and F-actin.

### Cx43-CT contains binding domains for tubulin, drebrin and the PDZ2 domain of ZO-1

**Fig 3:**
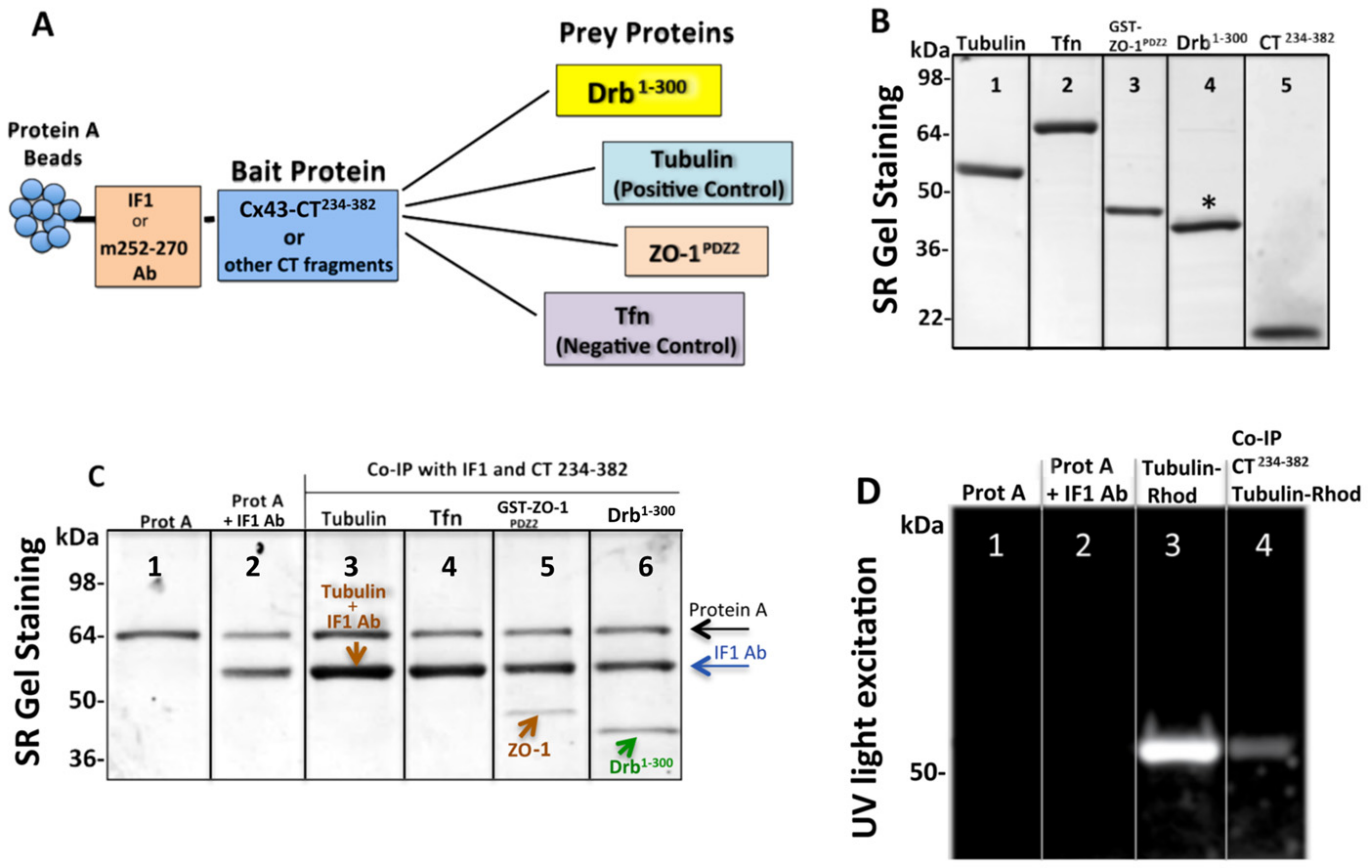
Connexin43 C-terminal region 234–283 contains binding sites for tubulin, drebrin and the PDZ2 domain of ZO-1. (A) Schematic of the co-IP strategy used to observe and quantify the binding of Drb^1-300^ to the Cx43-CT, using the latter as a “bait” and the other proteins (including positive and negative controls) as “prey”. (B) SDS-PAGE gel stained by SR showing the purity and expected molecular weight at: 55 kDa for tubulin, 80 kDa for transferrin (Tfn), ~43 kDa for GST-ZO-1^PDZ2^ tag, ~40 kDa for Drb^1-300^, and ~19.6 kDa for CT^234-382^. The asterisk above the Drb^1-300^ band points out Drb^1-300^ extra bands. (C) SDS-PAGE gel stained by SR after co-IP (representative lanes chosen as best example from four independent experiments) utilizing IF1 antibody and Cx43-CT^234-382^ as “bait”. Arrows indicate the protein A (*black*), IF1 heavy chain (*blue*), ZO-1 or tubulin (*brown*), and Drb^1-300^ (*green*). Note that in the co-IP with tubulin, the heavy chain of IF1 and tubulin bands overlap. (D) Unstained SDS-PAGE gel imaged by UV to detect tubulin-rhodamine after co-IP with Cx43-CT. Lanes 1 and 2 show the absence of non-specific binding of tubulin-rhodamine to protein A and the IF1 antibody respectively, and lane 3 shows the pure tubulin-rhodamine signal.

In previous studies Cx43 was pulled-down with drebrin E from a mouse brain homogenate, showing that these two proteins form a complex, however the existence of a direct interaction was not explored [15]. Here, we investigated the direct binding of the Cx43-CT to the drebrin N-terminal sequence (residues 1–300), which is part of the most conserved region between the embryonic and adult drebrin isoforms. The full-length Cx43-CT (residues 234–382) containing both the tubulin (residues 239–240 and 247–250) [12] and ZO-1 PDZ2 domain (ZO-1^PDZ2^) (residues 379–382) [55, 60] binding sites was chosen for these experiments. Purified ZO-1^PDZ2^ and tubulin were consequently used as positive controls for the co-IP, while transferrin (Tfn) protein was selected as a negative control due to its lack of any known association with Cx43 (Fig 3B). The GST tagged form of ZO-1^PDZ2^ was preferred as the ~43 kDa band was easier to detect and more intense than the ~18 kDa band for the untagged version. The co-IP was performed with two different Cx43-CT antibodies immobilized on protein A agarose beads: IF1 antibody raised against the 360–382 residues of Cx43-CT, potentially competing with the ZO-1^PDZ2^ binding domain [55, 61] or the m252-270 antibody (see schematic in Fig 3A). After protein hybridization and multiple washes, co-IP protein mixes were resolved by denaturing polyacrylamide gel electrophoresis (PAGE) and detected by SYPRO-Ruby (SR) gel staining. The results shown in Fig 3C were the most consistent and best representative lanes of at least four different replicates. Protein A band consistently showed at about 64 kDa (Fig 3C, lane 1), while the heavy chain of the IF1 antibody showed one band at 55 kDa (Fig 3C, lane 2). Since this band overlapped with the 55 kDa tubulin band (Fig 3C, lane 3), we used a rhodamine-conjugated tubulin that was detected by UV light (emission at 306 nm) (Fig 3D). The Tfn band was never present after co-IP, confirming that there was no Tfn/Cx43 interaction (Fig 3C, lane 4), while for both GST-ZO-1^PDZ2^ and Drb^1-300^ (untagged) we consistently detected bands at the expected molecular weights of ~43 and ~40 kDa, respectively (Fig 3C, lanes 5 and 6). Note that the Cx43-CT^234-382^ (19.6 kDa) band was not detected after co-IP because of the need of long electrophoresis to resolve the higher bands (excluding proteins under ~30 kDa). Similar results were observed when the m252-270 antibody was bound to the beads instead of the IF1 antibody. Taken together, these data indicated a direct binding event between the Cx43-CT^234-382^ and the Drb^1-300^ construct.

### Co-IP with Cx43-CT constructs reveals the binding sites for Drb^1-300^

**Fig 4:**
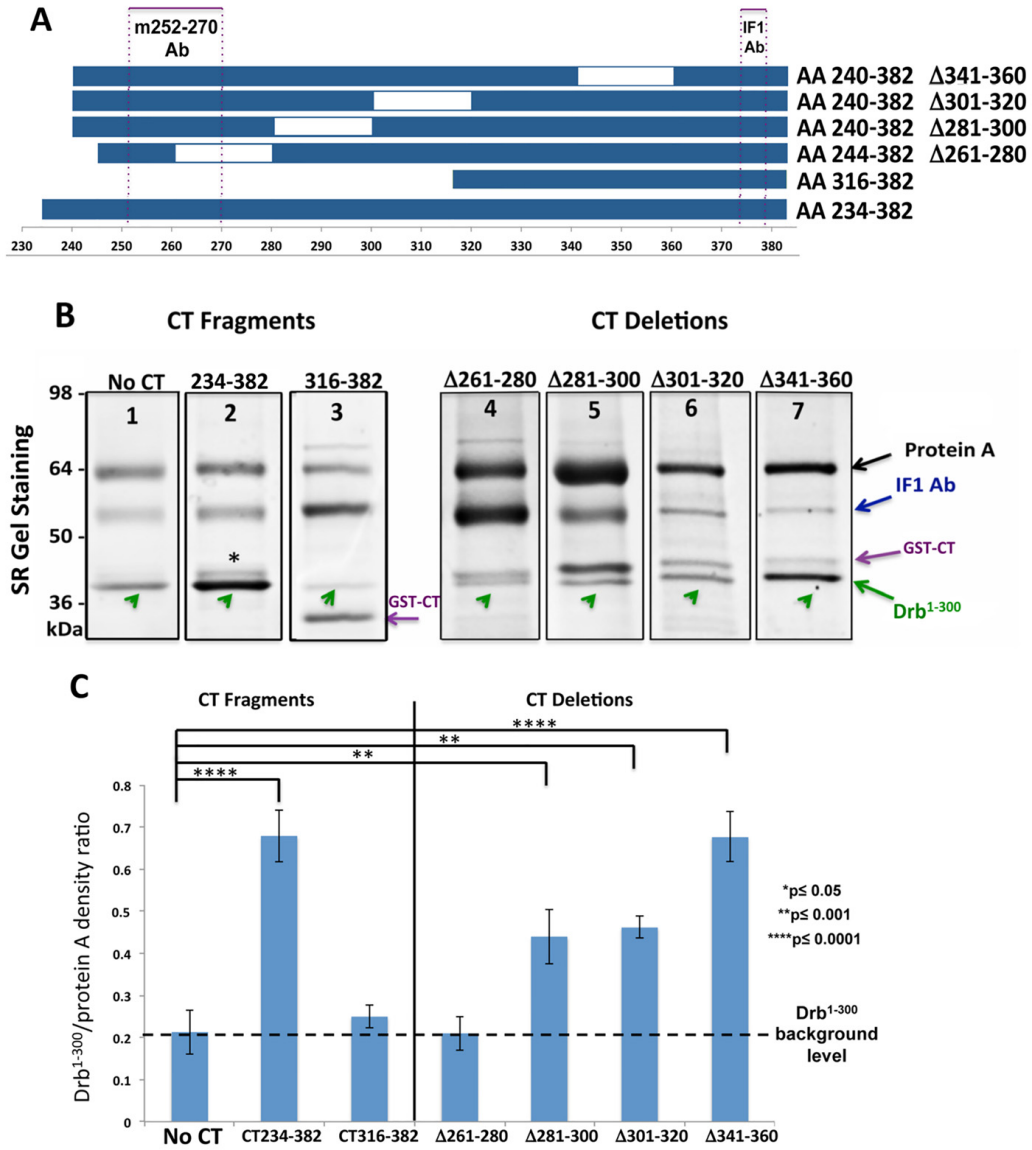
Co-IP reveals the Cx43-CT binding site for Drb^1-300^. (A) Schematic representation of the Cx43-CT constructs used to delineate the minimal domain(s) interacting with Drb^1-300^ by co-IP. IF1 and m252-270 antibody epitopes are represented by purple dotted boxes. (B) SDS-PAGE gel stained by SR after co-IP (representative lanes chosen as best example from four independent experiments). Arrows indicate the migration bands of protein A (*black*), the heavy chain of IF1 (*blue*), GST-CT constructs (*purple*), and Drb^1-300^ (*green*). The asterisk points out Drb^1-300^ extra bands. (C) Quantification of the data shown in (B). Drb^1-300^ co-IP band intensities were normalized to the intensity of the protein A band. The dashed black line indicates the Drb^1-300^ background level, providing the non-specific binding value. P values obtained through T-test are indicated. Error bars represent the standard deviation between four replicates.

To identify the minimal domain(s) of Cx43-CT involved in the interaction with Drb^1-300^, a co-IP strategy similar to that described Fig 3 was used in presence of a variety of GST-tagged Cx43-CT constructs presenting different lengths and deletions (Fig 4A). Since the binding sequence of the m252-270 antibody is not present in all the constructs, only the IF1 antibody was used (Fig 4A, dotted frames). Co-IP protein samples were resolved by SDS-PAGE and detected by SR gel staining. Results are shown in Fig 4B. The amount of non-specific Drb^1-300^ bound to the protein A agarose was estimated relatively to the intensity of the protein A band and used to normalize the efficiency of the binding (Fig 4C). After normalization and averaging the results from at least four different repeats, the full length Cx43-CT^234-382^ and Cx43-CT^240-382^ Δ341–360 constructs consistently showed the strongest binding (Fig 4B, lanes 2 and 7; graph in Fig 4C), while the Cx43-CT^316-382^ and the Cx43-CT^244-382^Δ261–280 (Fig 4B, lanes 3 and 4; graph in Fig 4C) presented a signal similar to the non-specific binding of Drb^1-300^ to the protein A agarose beads. These results indicate that the N-terminus portion of the Cx43-CT sequence (residues 240–316) is involved in the interaction with Drb^1-300^.

### Anti-Cx43-CT antibody m252-270 prevents Drb^1-300^/Cx43 interaction in co-IP experiments

**Fig 5:**
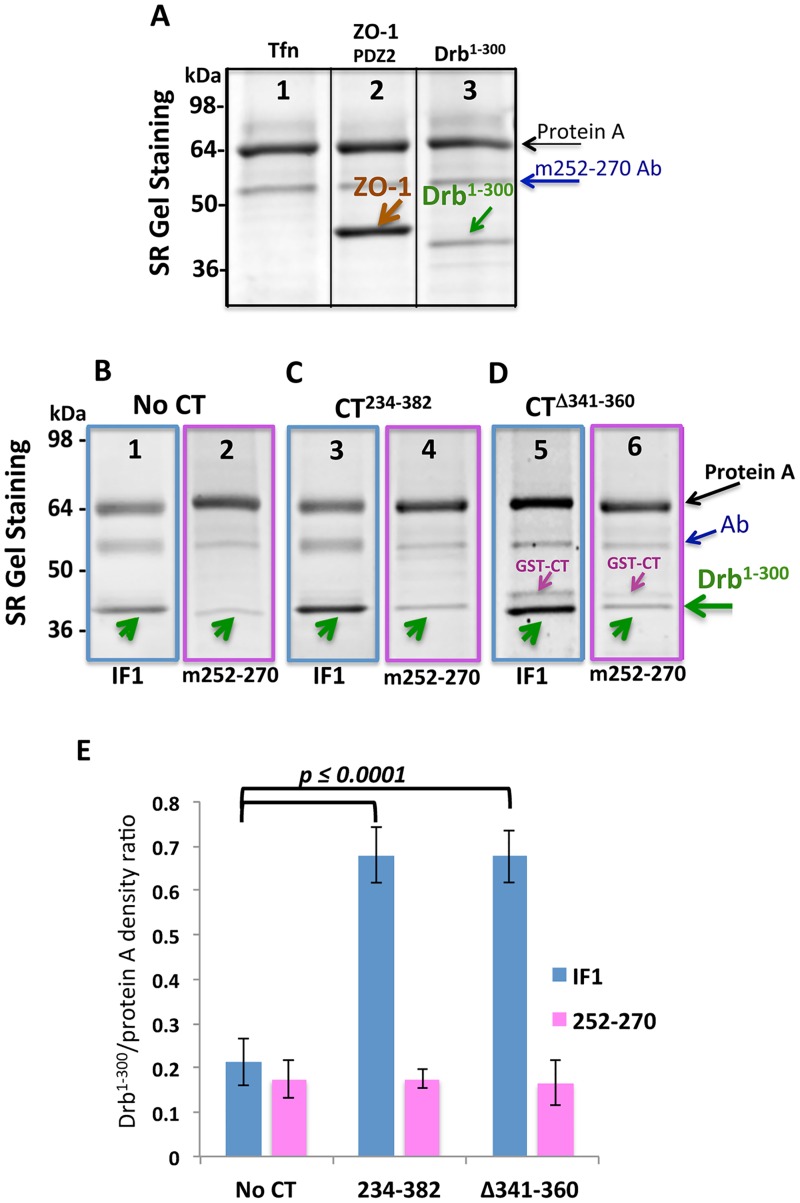
The antibody anti-Cx43-CT m252-270 inhibits Drb^1-300^ binding. (A) Representative lanes chosen as best example from four independent experiments of SDS-PAGE gels stained by SR after co-IP with full length Cx43-CT^234-382^ using the m252-270 antibody. Tfn and ZO-1^PDZ2^ were used as negative and positive controls, respectively (lanes 1 and 2). Arrows indicate the migration bands of protein A (*black*), the heavy chain of m252-270 (*blue*), ZO-1^PDZ2^ (*brown*), and Drb^1-300^ (*green*). (B-D) SDS-PAGE gel stained by SR of Drb^1-300^ co-IP, comparing the use of IF1 (lanes 3 and 5) and m252-270 (lanes 4 and 6) antibodies to immobilize the CT constructs. Arrows indicate the migration bands of protein A (*black*), the heavy chain of the antibody (IF1 or m252-270, *blue*), and Drb^1-300^ (*green*). (E) Drb^1-300^ co-IP band intensities were normalized to the intensity of the protein A band. P values obtained through T-test are indicated. Error bars represent the standard deviation between four replicates.

To further analyze what region of Cx43-CT is important for the interaction with Drb^1-300^, an antibody raised against the Cx43-CT residues 252–270 (m252-270 antibody) was tested. ZO-1^PDZ2^ and Tfn were utilized as positive and negative controls, respectively. As illustrated in Fig 5A, a very dense band corresponding to the GST-ZO-1^PDZ2^ domain was observed, confirming the interaction between the PDZ2 domain of ZO-1 and Cx43-CT^234-382^, and that m252-270 antibody is not inhibiting this binding. However, the intensity of the band detected for the Drb^1-300^ (Fig 5A, lane 3) was obviously very weak. This evidence supports the hypothesis that the m252-270 antibody prevents Cx43-CT/ Drb^1-300^ binding, by competing with Drb^1-300^. In order to confirm and quantify the existence of a binding competition between Drb^1-300^ and the mouse anti-Cx43 m252-270 antibody, we compared Drb^1-300^ band intensities after co-IP with IF1 or m252-270 antibodies, using the Cx43-CT^234-382^ or the Cx43-CT^240-382^ domain containing the 341–360 deletion. We chose these two Cx43-CT constructs that most strongly bind to Drb^1-300^, based on previous experiments (Fig 4).

As we described before (Fig 4B), the amount of Drb^1-300^ non-specifically bound to the protein A agarose beads was estimated and found to be similar for both antibodies (Fig 5B and 5E). Fig 5C and 5D represent the comparison between the IF1 and the m252-270 antibodies binding the CT^234-382^ fragment and the CT^341-360^ deletion. As confirmed by the quantification in Fig 5E, the co-IP with the m252-270 antibody eliminates the Cx43-CT/Drb^1-300^ binding, as shown by the Drb^1-300^ band intensities corresponding to the “No CT” level for both Cx43-CT constructs. This result confirmed the validity of our assay and at the same time indicated that the 252–270 Cx43-CT sequence contains a critical drebrin-binding site.

### NMR identifies the Cx43-CT residues involved in the Drb^1-300^ interaction

**Fig 6:**
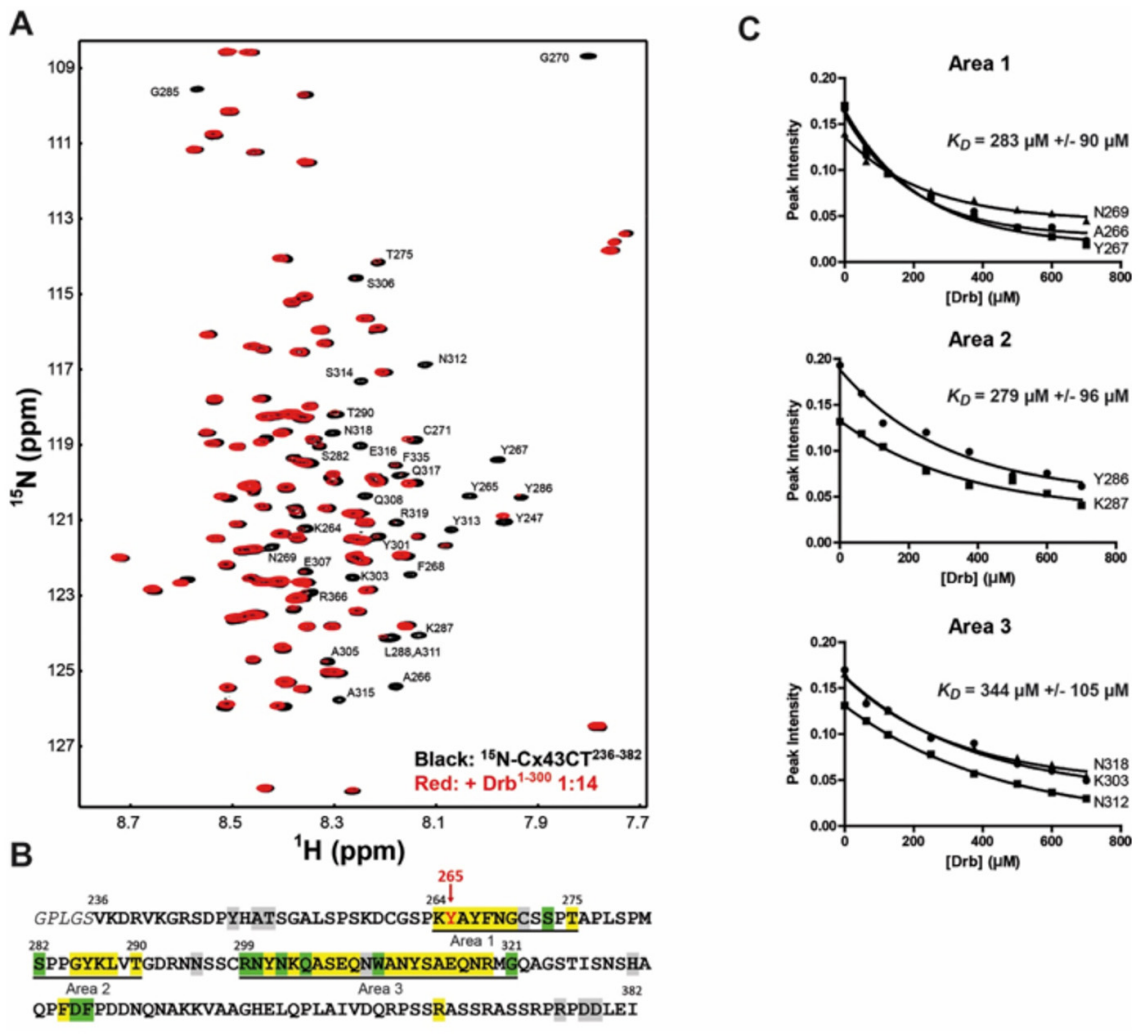
NMR identifies the Cx43-CT^234-382^ residues involved in the interaction with Drb^1-300^. (A) ^15^N-HSQC spectrum of Cx43-CT^234-382^ alone (*black*) has been overlaid with the spectrum obtained in the presence of the Drb^1-300^ construct at a 1:14 ratio (*red*). Residues affected by Drb^1-300^ are indicated in *black*. (B) Summary of the Cx43-CT^234-382^ residues affected by Drb^1-300^ are highlighted as follow: yellow, peaks broaden beyond detection; green, decreased intensity; grey, shift. (C) *K_D_* for each area of interaction was estimated by fitting the decrease in signal intensity of at least three affected residues. A subset of residues used for the calculation of the *K_D_* of each area is displayed.

Co-IP experiments suggest that Drb^1-300^ interacts with Cx43-CT in an area including residues 261–280, however the entire CT domain was needed to maximize the direct binding. To identify all the amino acid involved in the interaction and determine the *KD*, we performed an NMR titration. Unlabeled Drb^1-300^ was titrated into a 1X PBS solution containing ^15^N-labeled Cx43-CT^234-382^, at pH 7.5 (Fig 6A). The Cx43-CT residues affected by the Drb^1-300^ binding were mapped onto the Cx43-CT^234-382^ sequence (Fig 6B). Three areas of interaction were identified: 264–275, 282–290, and 299–321. The *KD* of the binding was determined by maintaining the concentration of the ^15^N-Cx43CT^234-382^ constant (50 μM), while adding increasing amounts of Drb^1-300^ from 50 to 700 μM (Fig 6C). The decrease of signal for a subset of residues from each area was fitted according to the nonlinear least squares method, providing a respective *KD* of 283 μM +/- 90 μM, 279 μM +/- 96 μM, and 344 μM +/- 105 μM. The *KD* values suggest Area 1 and 2 as primary binding site of interaction with Drb^1-300^ while the slightly weaker Area 3 could be a secondary site. This observation is in accordance with the co-IP experiments that identified the drebrin-binding domain around residues 261–280, and more specifically a primary site comprising residues 264–275. Since the Cx43-CT^240-382^Δ281–300 and Δ301–320 constructs do not completely restore the interaction with Drb^1-300^ (Fig 4), one could expect area 2 and 3 to be secondary binding sites needed for a higher binding affinity.

### Cx43-CT Y265D point mutation abolishes the Cx43/drebrin interaction

**Fig 7:**
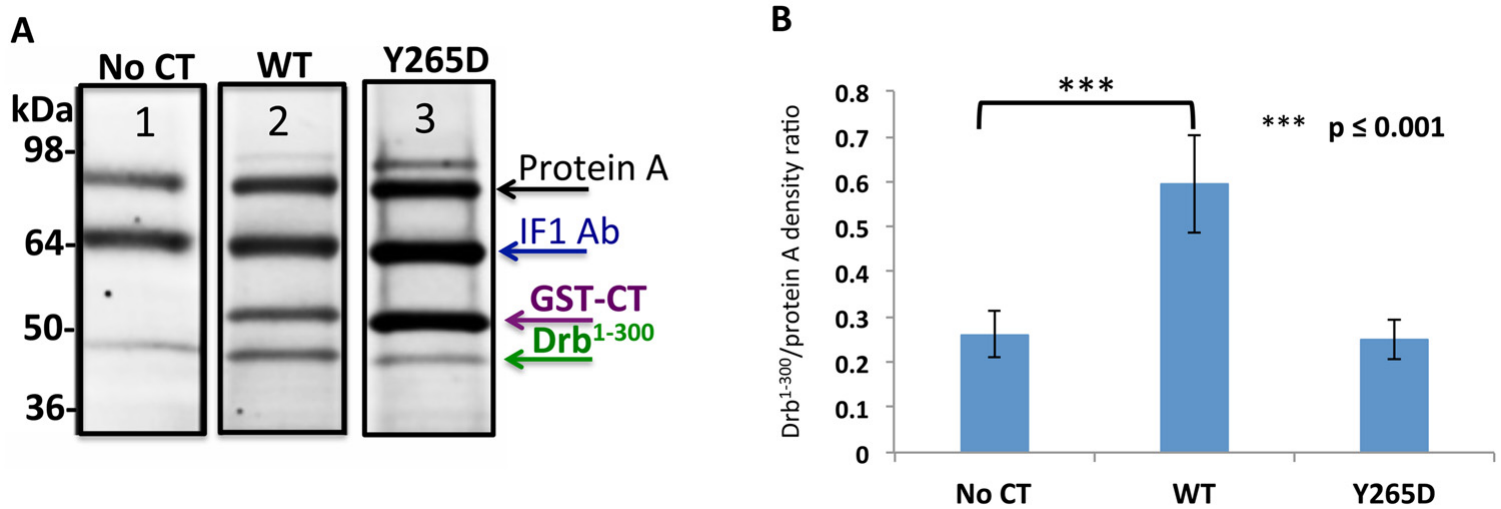
The point mutation Y265D abolishes Cx43/Drb^1-300^ binding. (A) SDS-PAGE gel stained by SR after co-IP (representative lanes chosen as best example from four independent experiments), where GST-Cx43-CT Y265D was used as a “bait” and Drb^1-300^ as “prey”. Arrows indicate the migration bands of protein A (*black*), the heavy chain of the IF1 antibody (*blue*), GST-Cx43-CT (*purple*), and Drb^1-300^ (*green*). Co-IP obtained with the GST-Cx43-CT WT is represented for comparison. (B) Quantification of the data shown in (A). Drb^1-300^ co-IP band intensities were normalized to the intensity of the protein A band. P value obtained through T-test are indicated. Error bars represent the standard deviation between four replicates.

Cx43 has been shown to associate and be phosphorylated by a number of kinases, including tyrosine kinases. Cx43 phosphorylation by Src at tyrosine residues 247 and 265 is well characterized and known for inhibiting gap junction communication [17, 62]. Interestingly, Y265 is located in one of the key domain of interaction with drebrin. We therefore investigated if Y265 could act as a regulative site for the Cx43/drebrin binding, with the hypothesis that once Cx43-CT becomes phosphorylated, this interaction would be inhibited. To answer this question, we designed a Cx43-CT^234-382^ construct with Y265D point mutation. The use of Asp substitution to study biological effects of phosphorylation is a widely used tool [8, 63, 64]. While the Cx43-CT^234-382^ wild type was able to pull-down Drb^1-300^ (Fig 7A), the band observed when using the Cx43CT-CT^234-382^ Y265D showed a density very similar to the “no CT” control (Fig 7B), indicating a loss of the Cx43-CT/Drb^1-300^ binding when the residue Y265 is phosphorylated.

**Fig 8:**
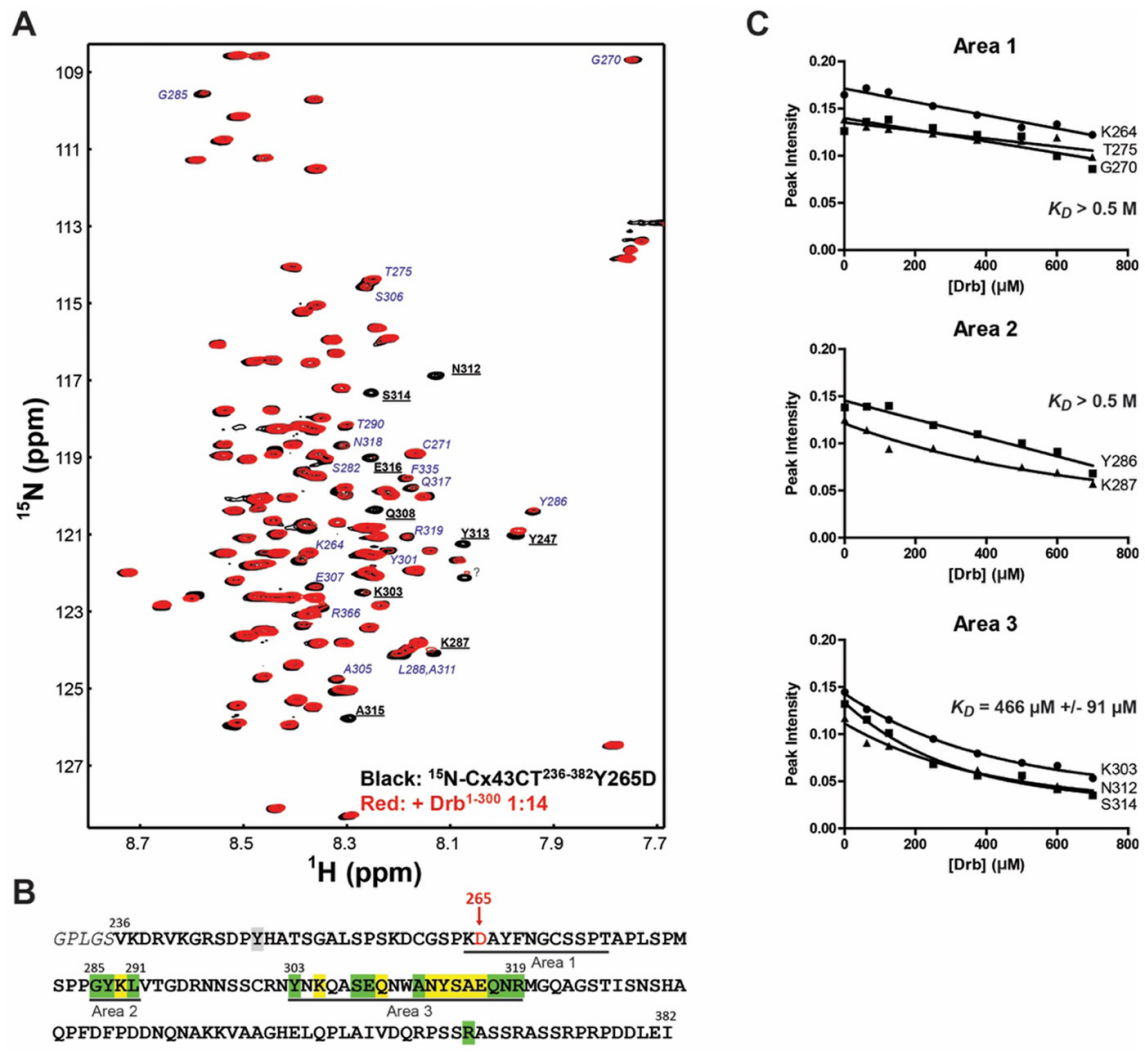
Cx43-CT^234-382^Y265D mutation inhibits the interaction with Drb^1-300^ by NMR. (A) ^15^N-HSQC spectrum of Cx43-CT^234-382^Y265D alone (*black*) has been overlaid with the spectrum obtained in the presence of the Drb^1-300^ construct at a 1:14 ratio (*red*). Residues affected by Drb^1-300^ similarly to the Cx43-CT^234-382^ WT are bolded and underlined in *black*, while residues weakly or no longer affected are indicated in *blue italic*. (B) Summary of the Cx43-CT^234-382^Y265D residues affected by Drb^1-300^ are highlighted as follow: *yellow*, peaks broaden beyond detection; *green*, decreased intensity; *grey*, shift. (C) *K_D_* for each area of interaction was estimated by fitting the decrease in signal intensity of at least three affected residues. A subset of residues used for the calculation of the *K_D_* of each area is displayed.

To confirm the inhibitory role of the Src Y265 phosphorylation on the Cx43-CT/Drb^1-300^ interaction, the Y265D phospho-mimetic mutant was used in an NMR titration experiment (Fig 8). Unlabeled Drb^1-300^ was titrated into the ^15^N-labeled Cx43CT^234-382^Y265D (Fig 8A). Significantly less residues were affected by the addition of Drb^1-300^ compare to the Cx43CT^234-382^ WT (Figs 6B and 8B). Importantly, the Y265D mutation completely abolished the interaction over Area 1 and 2 (*KD* measured > 0.5 M, Fig 8C), while the Area 3 showed a weak interaction with Drb^1-300^ with a *KD* around 0.5 mM. Combined together, these results implicate residues 264–275 in the Cx43-CT domain as critical for the interaction with the N-terminal portion of drebrin, as well as a strong potential for a regulation of this interaction by Src.

## Discussion

In this study, co-IP and NMR experiments were used to demonstrate that the Cx43-CT directly interacts with the highly conserved N-terminus region of drebrin. Three Cx43-CT areas were found to be involved in drebrin binding, with residues 264–275 being critical for the interaction. Mimicking Src phosphorylation within this region (Y265) significantly disrupted the binding between the Cx43-CT and drebrin. Immunofluorescence showed colocalization of Cx43, drebrin, and F-actin in astrocytes and Vero cells membrane, indicating that Cx43 forms a submembrane protein complex with cytoskeletal and scaffolding proteins. The co-IP data suggest that Cx43 indirectly interacts with F-actin through drebrin. Along with the known binding of the Cx43-CT with ZO-1 and tubulin, the data presented here further support the involvement of Cx43 in regulating cytoskeleton rearrangements, important for neuronal migration during brain development.

### Cx43 and drebrin colocalization in astrocytes and endothelial cells

In this study we show *in vivo* colocalization of Cx43, drebrin and F-actin in astrocytes and Vero cells membranes. During embryonic development in the brain, gap junctions provide intercellular communication between neural progenitor cells [26, 27]. In particular Cx43 and Cx26 are highly expressed at points of contact between radial glial and neurons [28, 65]. However, in the adult brain, Cx43 expression becomes restricted only to the astrocytes [30] where they form gap junctions between these cells and oligodendrocytes [66]. In astrocytes, Cx43 is also abundant at end-foot processes along blood vessels thus impacting the blood-brain-barrier [67]. Our rat brain analysis showed that Cx43 and drebrin colocalize in astrocytes and particularly in endothelial cells of the blood vessels, which form the blood-brain-barrier [68, 69]. In addition to providing a stable environment for neural function, the blood-brain-barrier also regulates specific channels and transporters to keep the ionic composition optimal for synaptic signaling function [68]. Connexins are involved in the regulation of vascular tone [70] and Cx43 has been detected on the membranes of porcine blood-brain-barrier endothelial cells by Nagasawa et al. [69]. These same authors [69] showed colocalization and interaction of Cx43 with the proteins occludin, claudin-5 and ZO-1 in tight-junctions of blood-brain-barrier. Furthermore, down regulation of connexins in astroglial weakens the blood-brain-barrier, which opens upon increased hydrostatic vascular pressure and shear stress. These results demonstrate that astroglial connexins are necessary to maintain blood-brain-barrier integrity [71].

Drebrin is one of the most abundant neuron-specific F-actin-binding proteins found in dendrites and is highly enriched in dendritic spines receiving excitatory inputs [72]. The drebrin-actin complex plays a crucial role in the regulation of dendritic spine morphology as the level of drebrin expression modulates dentritic spine morphology [40]. Drebrin is closely related to cognitive function, as many Alzheimer’s disease patients show significantly decreased drebrin mRNA levels in the cerebral cortex and hippocampus [73]. Golgi staining has revealed that the number of neuronal dendritic spines in the hippocampus is decreased in Alzheimer’s disease [74]. Thus based upon the study of Butkevich et al. (15) that drebrin is required for maintaining Cx43-containing gap junctions in their functional state at the plasma membrane, we would hypothesize that the decreased drebrin in Alzheimer’s disease causes increased degradation of Cx43 and consequently impairing cell-cell coupling.

Drebrin E is also found in several types of non-neuronal cells and is responsible for maturation of multiple organs during development and assuring their normal adult function by interacting with actin [34]. In COS-7 cells, increased expression of drebrin leads to formation of “spike” structures that also contain Cx43, while the absence of both these proteins leads to flatter membrane surfaces [54]. Here we confirmed that Vero cells and astrocytes show drebrin/Cx43/F-actin colocalization. While in Vero cells the three proteins overlap in correspondence of the opposing membranes between two cells, in astrocytes Cx43 and drebrin clearly colocalize inside of cellular spiky protrusions.

### Cx43 has a unique drebrin-binding site

Except for the binding of casein kinase 1 to phosphorylate Cx43-CT residues S325, S328, and S330, all the known Cx43-CT protein partners bind to the distal ends. For example, Giepmans and co-workers [17] first described that both α-and β-tubulin interact with a 35-amino acid juxtamembrane region in the Cx43-CT (residues 228–263). Microtubules have previously been identified as playing a critical role in Cx43 trafficking to the cell membrane [75].

Adjacent to this region (264–296) is a master regulatory domain that has known sites of phosphorylation and overlapping sequence motifs that enables binding with multiple molecular partners involved with Cx43 degradation (e.g. AP-2 and Nedd4). Conversely, the C-terminal end of the Cx43-CT is a binding site for the second PDZ domain of the scaffolding ZO-1 protein. This interaction has been described as being important for regulating the size of the Cx43 gap junction plaque in the cytoplasmic membrane [61, 76] and functionality [60, 77–80].

Here, we identified that drebrin not only interacts with the master regulatory domain, but also in a region with no known protein partner interactions (299–321). Interestingly, drebrin interacts with one of the two short α-helical domains (315–326) identified from the NMR solution structure [9]. The Cx43-CT α-helical domains undergo dimerization under acidic conditions [10] however, interaction with the protein partner Src and ZO-1 at the distal ends prevent dimerization. Although biological significance of dimerization is unknown, we speculate drebrin could inhibit dimerization by preventing the interaction of the α-helical domains.

### Cx43 interaction with the cytoskeleton is regulated by Src

**Fig 9:**
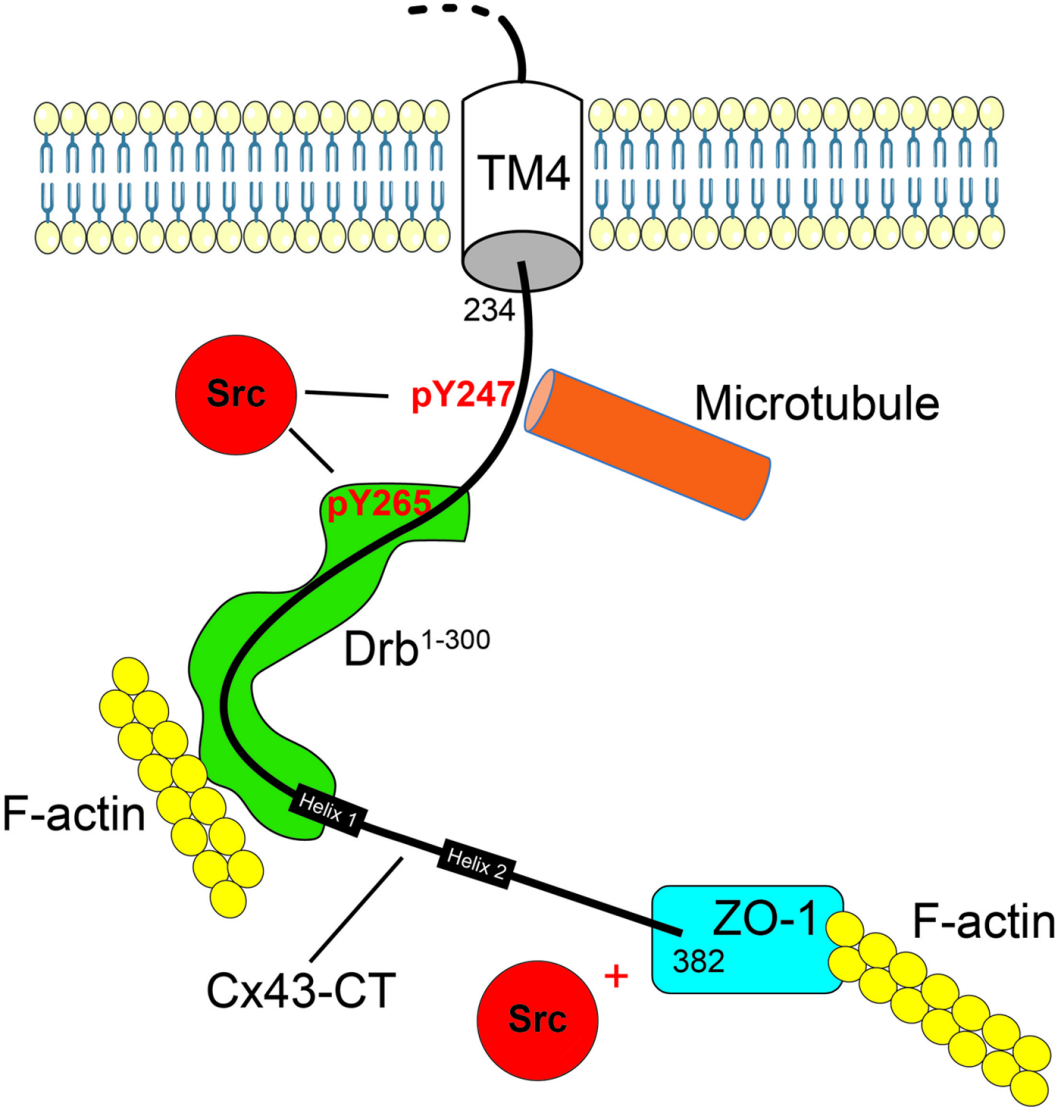
Model of drebrin interaction with Cx43 and the regulatory role of Src on Cx43 interaction with the cytoskeleton. Cx43-CT is represented as a *black line*, inserted in the membrane by its fourth transmembrane domain (TM4). The helical domains were indicated with *black boxes* and the Src phosphorylation positions were specified (*red*). The interacting proteins, Src (*red*), Drebrin (*green*), and ZO-1 (*blue*) have been schematized, as well as the cytoskeleton partners, microtubule (*orange*) and F-actin (*yellow*).

Here we found that posttranslational modification of Cx43 (Src-mediated phosphorylation) negatively regulates its interaction with drebrin. This provides new mechanistic details of the inhibition of Cx43-mediated cell-cell communication by Src. Src-induced phosphorylation of Cx43 has been correlated with channel closure [81]. Studies support a “particle-receptor” mechanism similar to that proposed for pH gating of Cx43 channels [82–84], which results in decreased electrical coupling by reducing the opening of channels and altering selectivity [85]. Our study presented here and those of other research groups, support an additional mechanism of Src to decrease gap junctional intercellular communication: the altering of Cx43 protein partners to enhance degradation (Fig 9). A commonality between the proteins that link Cx43 to the cytoskeleton is that Src can inhibit their interaction. For example, Cx43-CT residues Y247 and Y265 phosphorylated by Src inhibit the binding of tubulin [12] and drebrin, respectively. In the case of tubulin, at the gap junction plaque, this may be a mechanism in the disassembly process; at the trans-Golgi network, this may re-route trafficking to the plasma membrane (e.g., lateral membrane vs. intercalated disc) or inhibit trafficking to the plasma membrane, leading to increased intracellular proteasomal and/or lysosomal degradation [86]. For drebrin, depletion in cells results in impaired cell-cell coupling, internalization of gap junctions, and targeting of Cx43 for degradation [15].

While phosphorylation of the Cx43-CT by Src does not inhibit ZO-1 binding, we found that active c-Src can compete with Cx43 to directly bind ZO-1 [87]. Studies from the Gourdie and Lampe labs would suggest these transitions of Cx43 from the non-junctional plasma membrane into the gap junction plaque, and then through the degradation pathway(s) [88, 89]. Finally, Src activation also leads to indirect serine phosphorylation by Akt (S373), PKC (S368), and MAPK (S255, S279, and S282) that contribute to the lack of Cx43 at the plasma membrane. Akt may act in a similar manner as Src in that phosphorylation of S373 inhibits the Cx43 interaction with ZO-1 [89]. In addition, phosphorylation of S373 enables the binding of 14-3-3 leading to gap junction ubiquitination, internalization and degradation during acute cardiac ischemia [90]. Phosphorylation of S279/282 by MAPK increases the binding affinity by two-fold for the WW2 domain from the ubiquitin ligase Nedd4 leading to Cx43 gap junction degradation [91]. Finally, activation of PKC can halt the assembly of new gap junctions and its phosphorylation on S368 has been implicated in affecting gating and/or disassembly [92, 93] Altogether, the data point to Src playing a significant role in inhibiting Cx43-mediated cell-to-cell communication by altering channel gating (closing) and degradation (enhancing). Many different outcomes are caused by this single protein, and here we identified a relationship between Src and drebrin, which may help to better explain the very short half-life (1–5 hours) of connexins.

## Abbreviations

Cx43: Connexin43
Cx43-CT: carboxy terminus of Connexin43
Drb^1-300^: drebrin amino acids 1–300
CT^234-382^: Cx43-CT amino acids 234–382
CT^240-382^: Cx43-CT amino acids 240–382
ZO-1^PDZ2^: PDZ2 domain of ZO-1
SR: SYPRO Ruby
